# A Versatile Pep-CPDs Nanoprobe for Rapid Detection of mTBI Biomarker in Clinical Instances and Safe Fluorescence Imaging *In Vivo* for Improved Weight-Drop Mouse Model

**DOI:** 10.3389/fbioe.2022.807486

**Published:** 2022-03-07

**Authors:** Jian Shi, Xingmei Li, María José Cavagnaro, Jifeng Cai, Changquan Zhang, Na Li

**Affiliations:** ^1^ Center for Experimental Medicine, The Third Xiangya Hospital, Central South University, Changsha, China; ^2^ Department of Hematology and Critical Care Medicine, The Third Xiangya Hospital, Central South University, Changsha, China; ^3^ Department of Forensic Science, School of Basic Medical Sciences, Central South University, Changsha, China; ^4^ College of Medicine-Phoenix, University of Arizona, Phoenix, AZ, United States; ^5^ Department of Radiology, The Third Xiangya Hospital, Central South University, Changsha, China

**Keywords:** carbon polymer dots, non-toxic biosensors, mild traumatic brain injury, early-warning strategy, fluorescent imaging, TBI biomarkers, bioimaging, versatile functionalized biomaterial

## Abstract

Mild traumatic brain injury (mTBI) is the most common form of traumatic brain injury; however, it is the most difficult to be accurately identified in the early stage because it lacks more reliable biomarkers and detection methods. This study proposes a highly efficient system to detect a molecular biomarker for the early diagnosis of mTBI. The system was prepared by a lower cytotoxic peptide-modified fluorescent nanoprobe based on carbon polymer dots (pep-CPDs) with outstanding imaging capabilities. *In vitro* and *in vivo* tests were explored to the efficiency of pep-CPDs, inferring the good performances of cellular fluorescence imaging and *in vivo* imaging of mice. Moreover, an application of the versatile pep-CPDs on detecting the mTBI biomarker S100-β detection in a novel improved weight-drop mTBI mouse model and human blood samples has been successfully established. Overall, all these results indicate that the pep-CPD system is sensitive, rapid, non-toxic, and reliable for mTBI diagnosis compared with traditional detection methods. It shows a great potential in clinical and translational research and practical applications.

## Introduction

Mild traumatic brain injury (mTBI) is the most common type of traumatic brain injury. The World Health Organization (WHO) defines mTBI as a blow to or jolting the head, causing an acute brain function disorder, typically resulting in physical, cognitive, and/or emotional symptoms (Louie, 2010). Long-term complications often reported by patients with mTBI include increased anxiety, irritability, depression, and emotional lability, which had become an essential but latent clinical problem (Mulder et al., 2006; Jia et al., 2013; Xu et al., 2016). mTBI is often arduous to be accurately identified in the early stage due to untypical radiographic characteristics and imaging results, mild symptoms, and the lack of more reliable biomarkers and detection methods. These issues affect the authority and impartiality of judicial expertise as well as clinical diagnosis and treatment. Therefore, a rapid and accurate method for detecting the significant biomarkers of mTBI is very crucial and indispensable.

Over the last few years, in the wake of clinical neurology, material chemistry, and molecular diagnostics, biomarkers have been proven to have a significant value in the early and rapid diagnosis of mTBI treatment. S100 calcium-binding protein-β (S100-β), primarily produced by astrocytes in the central nervous system, is considered as an essential early biomarker of mTBI (Rahimian et al., 2020). In the previous studies, Rahimian and Çevik have found that S100-β was more sensitive and appeared during the early phase of mTBI, which was more befitting for the early diagnosis of mTBI than other biomarkers (Vos et al., 2010; Thelin et al., 2013; Çevik et al., 2019; Rahimian et al., 2020). In addition, a meta-analysis has shown that S100-β routine examination in hospitals can significantly reduce the frequency of radiologic studies for mTBI patients (Oris et al., 2018).

At present, the detection of S100-β still depends on the traditional enzyme-linked immunosorbent assays (ELISAs), chemiluminescent immunoassay, magnetic bead-based methods, and so on (Smit et al., 2005; Kim and Searson, 2015). However, those assays are not particularly satisfactory in practical applications due to their various shortcomings, such as false-positive rates, the cost of the instruments, and complicated and time-consuming procedures. Moreover, the increasing number of mTBI cases worldwide implies the development of new efficient and easy diagnosis tools (Vos et al., 2010; Han et al., 2020; Pankratova et al., 2021). Therefore, a novel nanofluorescence sensing detection method based on carbon dots (CDs) has emerged as the times require (Xia et al., 2019). In recent research and related literatures, as a novel type of CDs, carbonized polymer dots (CPDs) have gained more attention due to their convenient synthesis procedure, excellent biocompatibility, and nontoxic characteristics (Tao et al., 2019). Combining these advantages with excellent water solubility, CPDs are anticipated as a kind of fluorescent probe in biomedicine, sensing, bioimaging, and diagnostic applications such as in the real-time monitoring of apoptosis and selective imaging of bacteria (Zhao et al., 2019; Ru et al., 2020; Zhao et al., 2020).

**SCHEME 1:**
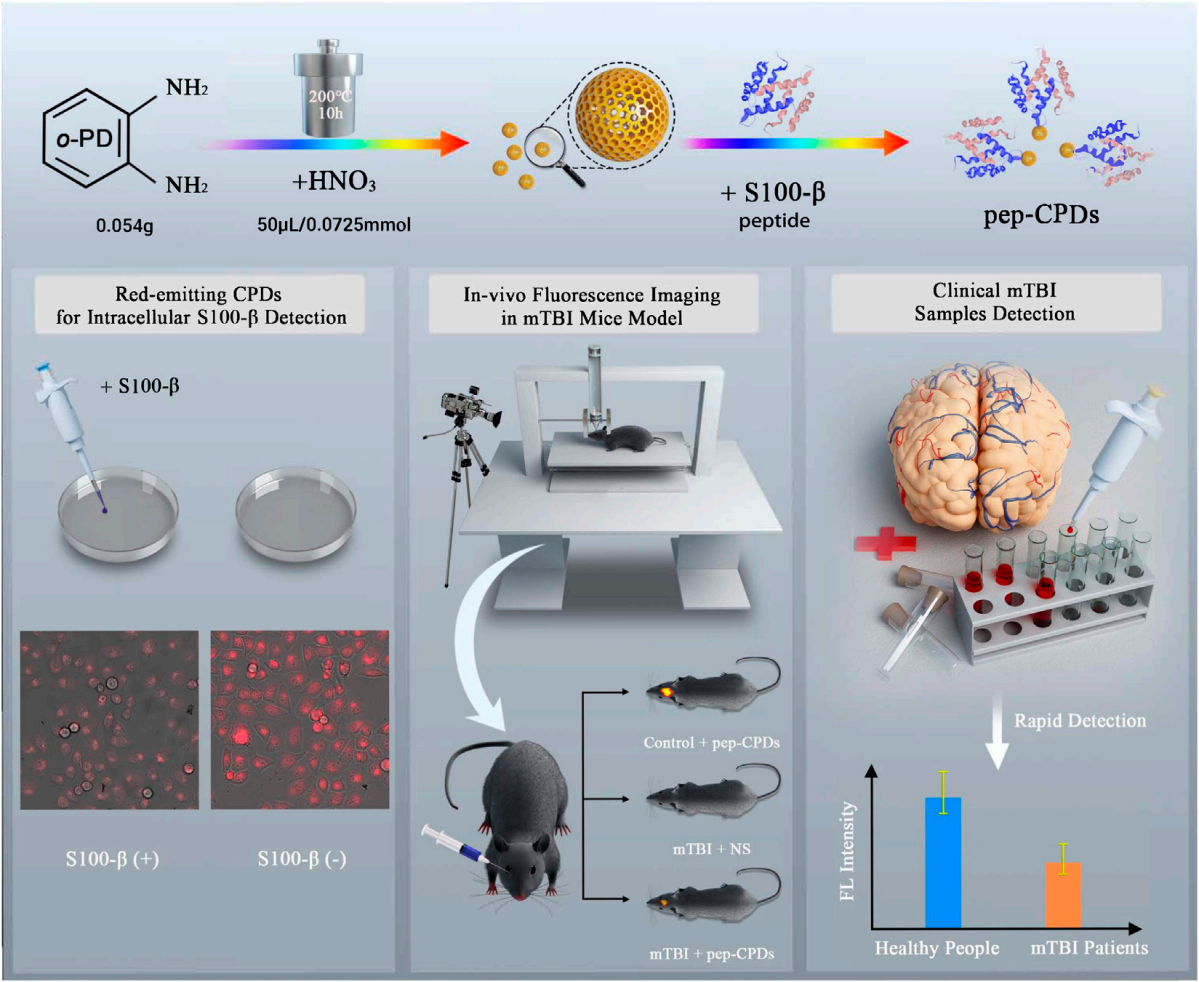
Schematic illustration of CPD synthesis and the peptide-based fluorescent nanoprobe for S100-β detection as the predication of mTBI.

In this study, we developed a rapid and sensitive facial one-pot synthesis of peptide-modified fluorescent nanoprobe based on CPDs (pep-CPDs) *via* a hydrothermal system (Scheme 1) for detecting the important mTBI biomarker S100-β in *in vivo* and *in vitro* imaging. The safety and low cytotoxicity of pep-CPDs were proven by the LDH assay, MTT assay, cellular imaging, and acute toxicity test in mice. The effectiveness of pep-CPDs on detection was demonstrated by predicating S100-β in mTBI patients’ blood samples. An improved weight-drop injury mechanical device (closed head injury apparatus) was designed and used to construct the mTBI animal model. Then, we completed the *in vivo* imaging detection in mice modeling with pep-CPDs. Finally, we explored the applicability of the pep-CPD detecting system in mTBI clinical instances to verify this versatile pep-CPD nanoprobe’s authenticity and dependability.

## Materials and Methods

### Materials

NHydroxysuccinimide (NHS, 99%), N3-dimethylpropane-1,3-diamine (EDC, 99%), o-phenylenediamine (o-PD, 99%), HNO3 (GC, 70%), and bovine serum albumin (BSA, molecular biology grade) were purchased from Sigma-Aldrich (Shanghai, China). The S100-β peptide NH2-TRTKIDWNKILS was synthesized and purified by GL Biochem (Shanghai, China). S100-β, GFAP, UCH-L1, Tau, MPB, serum amyloid A (SAA), ethanediamine (AR 97%), lysine (BC grade), citric acid (reagent grade | purity ≥99.5%), and glucose (biotech grade | purity ≥99.5%) were obtained from Sigma-Aldrich (Shanghai, China). Ultrapure water (18.2 mΩ cm^–1^) was used to prepare all solutions.

### Apparatus and Reagents

An F-4700 fluorescence spectrophotometer (catalog number; Hitachi Co., Tokyo, Japan) was used to record emission spectra. Both the slit widths of excitation and emission were 5 and 10 nm, respectively. The UV-visible (UV-Vis) spectra were recorded on a UV-Vis spectrophotometer (Shimadzu, UV-2450). The FEI Tecnai G2 60–300 microscopy (Hillsboro, OR, United States) was used to capture transmission electron microscopy. The zeta potential was determined by the Zeta-sizer Nano ZS instrument (Malvern Inc., United Kingdom). The chemical structure and functional groups on the CPD surface were confirmed using a Fourier transform infrared spectrometer FTIR-850 (Tianjin Gangdong Sci. & Tech. Co., Ltd., China). Cell imaging was performed using a laser scanning confocal microscope (ZEISS Cell discoverer 7 with LSM 900; Germany). The quantum yield (QY) measurements of CPDs were tested by an Edinburgh FS920P fluorescence spectrometer equipped with an integrating sphere. The QYs were calculated by fluorescence software and repeated three times.

### One-Pot Synthesis of Water-Soluble Fluorescent Carbon Polymer Dots

The CPDs were synthesized by a hydrothermal method according to a previous protocol described in the literature, with slight modifications (Liu et al., 2018). Briefly, 0.054 g o-PD was dissolved in 10 ml of deionized water and then 50 µl (0.725 mmol) HNO_3_ were added. After stirring at room temperature for 5 min, the solution was transferred to a poly Teflon-lined autoclave (25 ml) and heated in an oven at 200°C overnight. Then, the reaction mixture was cooled to room temperature. The dark-blue solution was obtained after filtration with 0.22 µm polyethersulfone membrane to remove large particles and dialyzed in a 500 Da dialysis bag against DI water. Then, the CPD solution was lyophilized for further use.

### Fluorescence Sensing of S100-β Using Pep-Carbon Polymer Dots

The NH_2_ groups of S100-β peptide were conjugated to the COOH groups on the surface of CPDs in the presence of coupling agents (EDC and NHS). Briefly, 3.5 mg EDC and 5 mg NHS were added into the 1 mg/ml CPD solution, followed by stirring for 30 min. Then, 100 μl of 1 mg/ml S100-β peptide was added to the mixture and reacted for 30 min at room temperature. The obtained CPDs conjugated with peptide are referred to as pep-CPDs. Further purification of the pep-CPDs was conducted to remove unbound peptide and unreacted chemicals through a dialysis bag (MWCO 500–1,000 Da) for 1 day in the dark. Distilled water (500 ml) was changed at least three times with 6 h intervals.

For S100-β detection, aqueous solutions of S100-β with different concentrations (1–10 ng/ml, 30 μl (Vos et al., 2010)) were prepared, which were then added separately to aqueous solutions of pep-CPDs (1 mg/ml, 70 μl). The corresponding fluorescence spectra were added to assess the dependence of fluorescence intensity on the concentration of S100-β. For the selectivity study, the fluorescence response toward ethanediamine, lysine, citric acid, BSA, GFAP, UCH-L1, Tau, MPB, SAA protein, glucose, and were tested in the same way. The fluorescence signal response of the reaction system was detected by a F-4700 fluorescence spectrophotometer, where the excitation wavelength was 520 nm, and the emission wavelength was ranged from 600 to 750 nm.

### Detection of S100-β in Serum

Serum samples were collected from the patients with a mean age of 27 years (range, 23–30 years) enrolled in the current study, and all the patients had no underlying severe hypercerebral hemorrhage before sample collection. After clotting, serum samples were isolated by centrifugation at 4,000 *g* for 15 min at 4°C. The serum was separated into aliquots and stored at −80°C. A standard addition method was adopted to detect the S100-β in serum.

### Cellular Preparation

HeLa cells was a gift of Professor Jifeng Cai in Changsha, China, and cultured in 24-well plates for 24 h (at a concentration of 5 × 10^4^ cells per well). The cultured cells were washed twice in PBS, then followed by adding 250 μl of fresh medium containing various concentrations of pep-CPDs. The cells were incubated in a humidified environment for an additional 24 h (37°C in a 5% CO_2_ atmosphere).

### *In Vitro* Cell Viability

The viability of the cells was determined as follows. Before adding 1 ml of the MTT stock solution (500 mg/ml), the cells were washed with PBS buffer two times. Then, they were co-incubated with different concentrations of 10 μl pep-CPDs and an MTT solution for 4 h under the same conditions. Next, the medium was removed; 100 µl of DMSO was added into each well. The plate was gently rotated on an orbital shaker for 10 min to completely dissolve the precipitation. Finally, the optical density (OD) of the cell suspension was measured at 520 nm using a microplate reader (Thermo Scientific™/Multiskan Sky). Each condition was repeated three times. The cell viability (%) was determined as the absorbance ratio between the cells after various treatments and the control cells without treatment. For the LDH assay, the CyQUANT™ LDH Cytotoxicity Assay Kit (Thermo Fisher™) was used, with the same number of cells and preparation process as in the MTT assay. After incubating with different concentrations as 10 μl pep-CPDs for 4 h, the LDH reaction mixture was added. After a 30-min incubation, the reaction was terminated by adding a stop solution and absorbance was measured using a microplate reader. The LDH release percentage was calculated and converted based on the manufacturer’s instructions.

### Mild Traumatic Brain Injury Experimental Device and Measurement System

The experimental device and measurement system were developed especially for this study. The equipment’s main body was an improved weight-drop impactor based on Professor M.A. Flierl’s classical TBI model (Flierl et al., 2009), newly produced and improved by Professor Na Li from our research team. In our improved equipment, new speed limit grating and high-speed camera are used to ensure that the injury is accurate and quantitative and to keep the injury homogeneous and controllable, which produces clear motor and cognitive deficits that were typically seen after TBI (Liu et al., 2015). It utilized a stereotaxic device to immobilize the animal (rat/mouse) and ensures a repeatable alignment with the impact device. For the experiment, male, pathogen-free C57 mice (10–12 weeks, 25–28 g) were selected. The mice were anesthetized with inhalative isoflurane using a standard anesthesia machine. Then, the approximate surface display of junction of the sagittal and coronal sutures at the top of the skull was visually identified and the area of impact was located. Then, a drop tube with the impact shaft was loaded with the weight of 300 g. After the activation of the startup switch, the impact shaft was released from its securing drop tube and did a free fall onto the mouse head. Then, all mTBI mice were taken care of; they were evaluated through the mTBI animal modeling effect by NSS (neurological severity score) and beam balance tests with statistical analysis. Other processes were kept consistent with Professor M.A. Flierl’s TBI protocol. Our improved mechanical device is delivered to the intact dura *via* an adjustable electromagnetic-driven piston (impactor) of variable diameter, thus providing a more localized injury with a velocity control grating and real-time photography system, which could not only ensure model integrity but also help to improve the model quality by providing design suggestions and best practices.

### *In Vitro* Fluorescence Imaging

HeLa cells were seeded in a 6-well plate at a density of 10^5^ cells per well and cultivated in an incubator at 37°C for 24 h. Cells were treated with fresh culture media containing 10 μl of 300 μg/ml concentration pep-CPDs and incubated for 1 h. Cells were then washed twice with PBS (pH 7.4) and observed under a confocal microscope for capturing the location of pep-CPDs.

### Fluorescence Imaging *In Vivo* and *Ex Vivo* of C57 Mice

All procedures involving mice were conducted humanely and performed by trained and experienced personnel. All studies involving rodents were reviewed and approved by the Third Xiangya Hospital (IRB No.2020-KT58). Six-week-old male/female C57 mice were purchased from the Department of Laboratory Animals, Central South University. After the mice were anesthetized with diethylether, 300 μl of pep-CPDs and normal saline were injected into their inner canthus for a higher injection success rate and better drug distribution. The fluorescence images of the brain area were obtained by a small animal *in vivo* fluorescence imager (IVIS Lumina II, Caliper Life Sciences), using the excitation wavelength of 480 nm and the reception wavelength of 660 nm.

### Statistical Analysis

Statistical analysis was performed using the statistical software IBM SPSS 19.0, and the measurement data were analyzed by mean ± standard deviation.

## Results and Discussion

### Principle of Mild Traumatic Brain Injury Diagnosis With the Fluorescent Sensing Pep-Carbon Polymer Dots Nanoprobe

The schematic illustration for preparing CPDs and the biological application *in vitro* and *in vivo* were shown in Scheme 1. Firstly, The CPDs were obtained with a red emission of 640 nm *via* a one-pot hydrothermal method, referring to Liu’s method (Liu et al., 2018). The amino-modified S100-β peptides were conjugated onto the surfaces of CPDs by using the amino-carboxyl reaction, while the peptide-modified CPDs retain higher affinity to the target S100-β (Flierl et al., 2009), which allows a sensor operation at low S100-β concentrations. The high affinity of pep-CPDs toward S100-β could be the interaction between S100-β and peptides on the surface of CPDs, causing the aggregation of pep-CPDs (Liu et al., 2015). Therefore, the fluorescence intensity of pep-CPDs at 640 nm is gradually decreased along with the increasing concentration of S100-β, which may be attributed to the aggregation-induced emission quenching. Furthermore, there was distinct fluorescence between the HeLa cells with S100-β spiked and without, in cellular fluorescence imaging. It was noted that an improved weight-drop mouse model was constructed for the mTBI diagnosis. *In vivo* imaging was applied to the pep-CPDs, and the results showed different signals between the mTBI mouse group and the control group. Besides, a rapid and sensitive fluorescent detection of S100-β in clinical samples can be achieved in terms of the pep-CPDs. Overall, the described pep-CPDs exhibited outstanding performances for capturing S100-β in different biological samples, offering a novel avenue for the early diagnoses of mTBI.

### Characterization of Pep-Carbon Polymer Dots

Water-soluble pep-CPDs were prepared in a one-step hydrothermal reaction performed in an autoclave at 200°C for 8 h. As presented in Supplementary Figure S1A, transmission electron microscopy was used to visualize the structure characterization of the prepared CPDs with an average size of 5.1 nm. The FTIR spectrum of the CPDs had an apparent peak around 2,965 cm^−1^, indicating the presence of -OH groups and a peak at 1,407 cm^−1^ showing the presence of the C=O group (Wang et al., 2020; Yang et al., 2020; Liu et al., 2021) (Supplementary Figure S1B). The peak at 1,640 cm^−1^ corresponded to c = c starching vibration. These data revealed that the synthesized CPDs are rich in carboxylic groups, which helped to combine the S100-β peptide in the next experiments. The aqueous solution of CPDs was measured by zeta potential with a negative charge (-13.4 mV) (Supplementary Figure S2A). After conjugation with the S100-β peptide, the zeta potential changed from −13.4 to −3.2 mV, indicating a successful peptide conjugation on the CPDs’ surface. Interestingly, the CPDs conjugated with peptide still presented a negative overall zeta potential, which is essential to avoid a fast removal of the particles from blood circulation for biology experiments.

### Optical Properties of Pep-Carbon Polymer Dots

**FIGURE 1:**
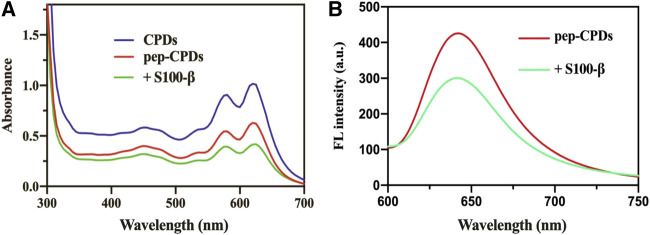
**(A)**. UV-Vis absorbance spectrum of CPDs (blue line), pep-CPDs (red line), and its addition of S100-β (green line) **(B)**. Fluorescence spectra of pep-CPDs in the presence and absence of S100-β. λex = 520 nm.

The UV-Vis absorption and fluorescence emission spectra of CPDs dispersed in water were recorded at room temperature. As shown in Figure 1A, the UV-Vis absorption spectrum of CPDs displays several absorption bands between 450 and 630 nm, which were in agreement with the previous work (Liu et al., 2018). The peptide was self-assembled onto CPDs *via* π-interactions and the amino-carboxyl reaction, and it will not affect the general functionality such as the physical and chemical properties of the surface (Joshi et al., 2020). After the addition of the target S100-β, it was presented that the absorbance value decreased compared to the pep-CPDs (Figure 1A, green line). Moreover, the fluorescence emission spectra of CPDs presented a fluorescence intensity peak at 640 nm with an excitation of 520 nm (red curve in Figure 1B). Once the target S100-β was added in the system, fluorescence quenching was induced because pep-CPDs were highly combined with S100-β (blue curve in Figure 1B). In addition, the QY of the as-prepared CPDs was calculated to be approximately 4.1%. These results proved that the as-prepared nanoprobe has excellent optical performance and is capable of the fluorescence detection of S100-β.

### Optimization of Experimental Parameters

To acquire more sensitive S100-β detection, we optimized different experimental parameters, including the concentration of the S100-β peptide and response time. As presented in the Supplementary Figure S2B, the relative fluorescence change increased upon raising the concentration of S100-β peptide from 0.1 to 1 mg/ml, and started to level off till 1 mg/ml. Thus, a peptide concentration of 1 mg/ml was used for the S100-β detection in the following experiments. Another factor that affects the detection efficacy of the sensing system is the response time. After being treated with 100 ng/ml S100-β, the fluorescence of pep-CPDs gradually faded away within 10 min (Supplementary Figure S2C). The pep-CPDs toward S100-β detection were taken in 10 min to achieve a remarkable signal output faster than the reported S100-β-based method that usually required 0.5–6 h (Wang et al., 2014; Kim and Searson, 2015).

### The Specificity of the Pep-Carbon Polymer Dots Against S100-β

**FIGURE 2:**
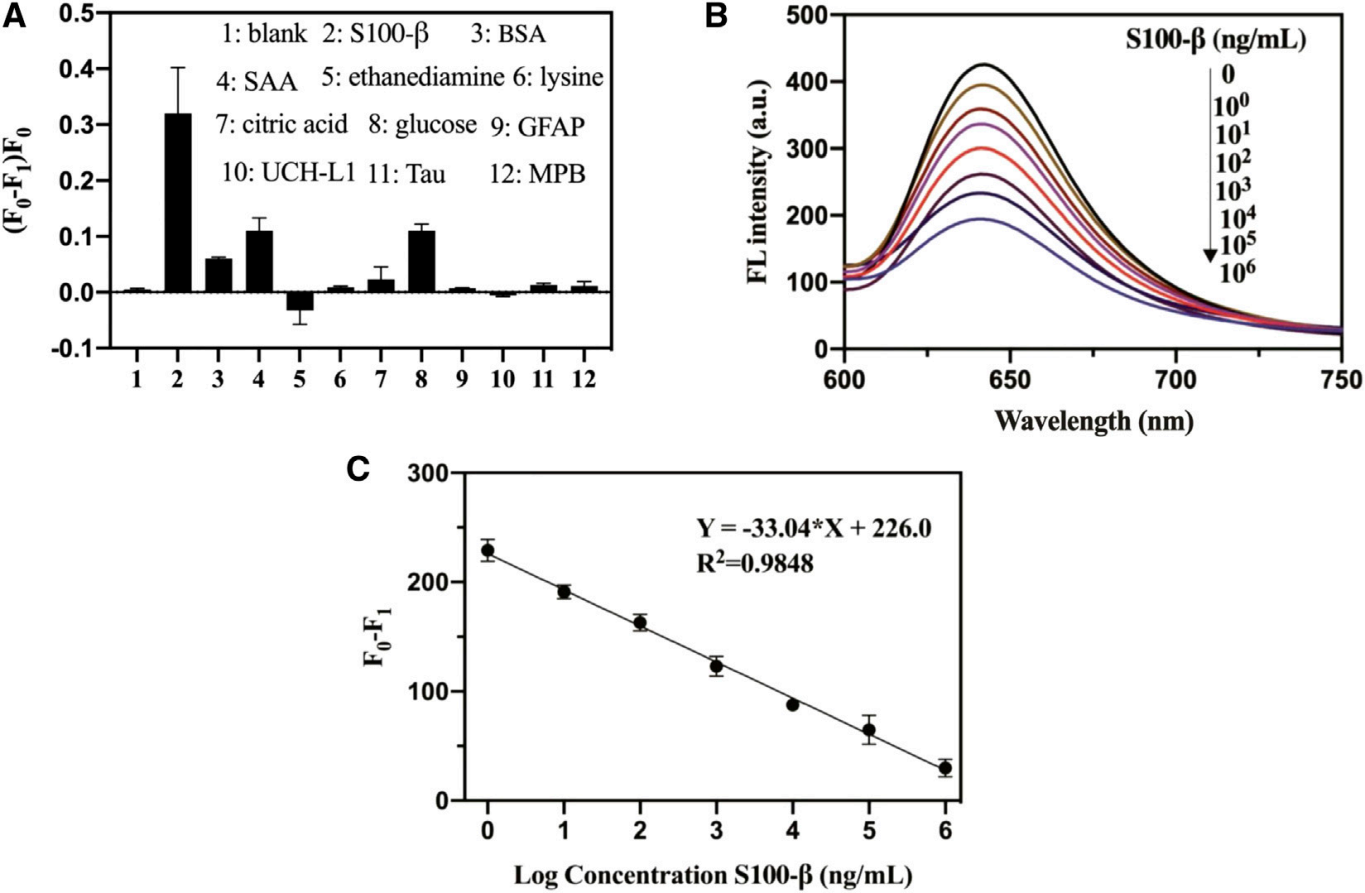
**(A)**. Specificity of pep-CPDs for S100-β over interferents. The concentrations of S100-β and interferents were 10^3^ ng/ml. **(B)**. Relationships between fluorescence intensity of pep-CPDs and S100-β from 0 to 10^6^ ng/ml. **(C)**. Calibration curve of the related fluorescence intensity (F0-F1) versus the concentration of target S100-β. F_0_ and F_1_ represent the fluorescence intensity of the nanoprobe in the absence and presence of target S100-β.

To evaluate the specificity of the S100-β sensing assay, we further performed several control experiments. As presented in Figure 2A, the interfering protein (BSA and SAA protein, GFAP, UCH-L1, Tau, and MPB) exhibited a small fluorescence change. No distinct difference was observed between interfering molecules after using the test ethanediamine, lysine, citric acid, and glucose, whereas upon the addition of S100-β, the relative fluorescence changes of pep-CPDs were the highest among these molecules. Therefore, the pep-CPD nanoprobe has been demonstrated to be useful for the specific detection of S100-β.

### Analytical Performance of Pep-Carbon Polymer Dots Toward S100-β

The fluorescence quenching degree of the pep-CPDs depends on the target S100-β concentration (Figure 2B). The corresponding plot of the fluorescence response as a function of S100-β concentrations was revealed in Figure 2C. It displayed good linearity with the concentrations of S100-β range from 1 to 10^6^ ng/ml (F_0_ and F_1_ represent the fluorescence intensity of the nanoprobe in the absence and presence of target S100-β). The linear regression equation of S100-β was described as Y = −33.04*X + 226.0 (*R* ^2^ = 0.9848). A limit of detection (LOD) of 0.1 ng/ml was calculated based on a signal-to-noise ratio (S/N = 3). Notably, the whole detection process can be completed within 20 min, which is significantly beneficial to rapid detection in clinical instances.

### Fluorescence Imaging of S100-β in Living Cells

**FIGURE 3:**
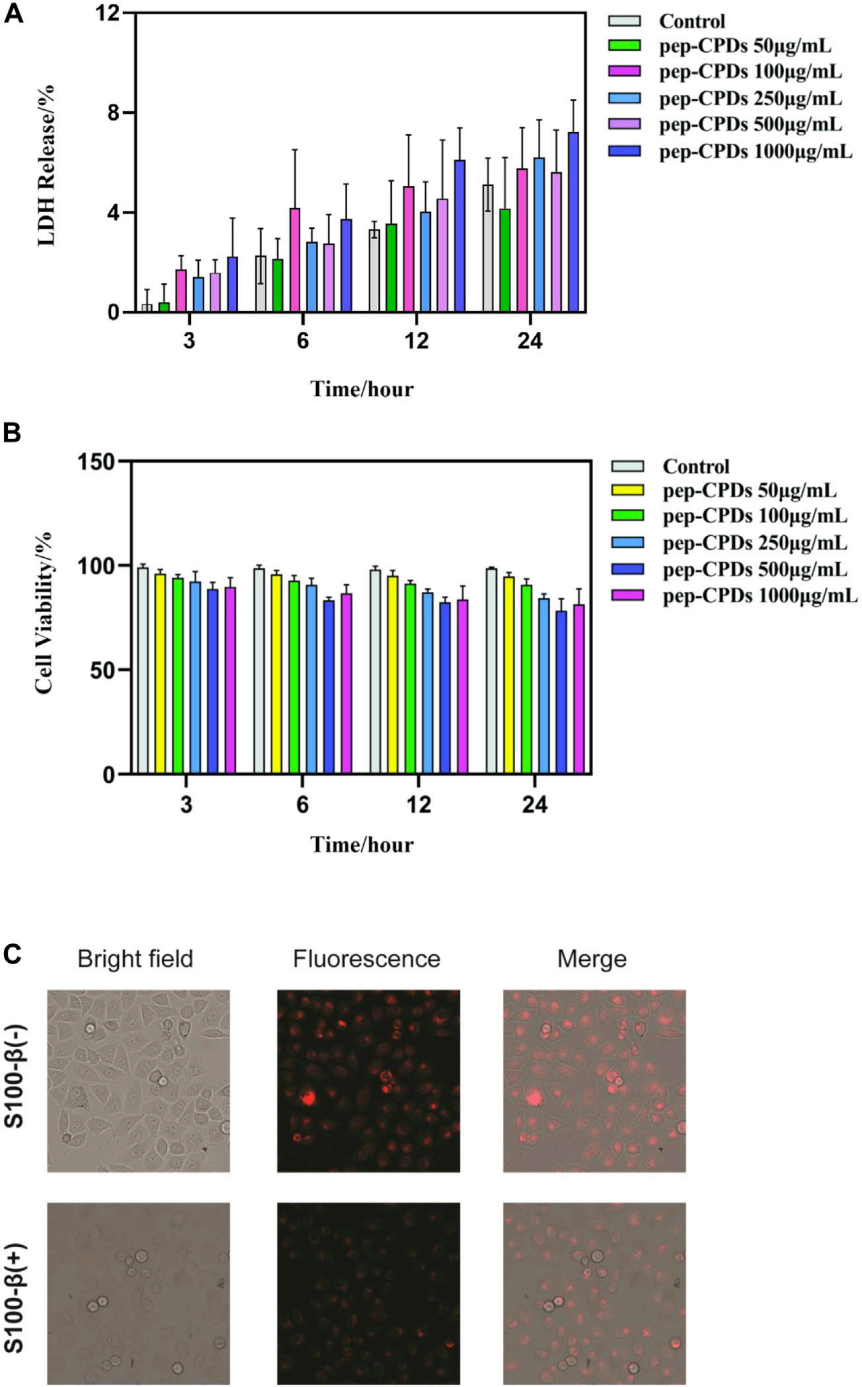
The evaluation of CPDs effect on *in vitro* cell viability and its application in the cellular fluorescence imaging. **(A)**. HeLa cells were treated with various concentrations of pep-CPDs (right panel) for 3, 6, 12, and 24 h. The LDH activity in cell supernatants or lysates was analyzed by the CyQUANT™ LDH Cytotoxicity Assay Kit (ThermoFisher) according to manufacturers’ protocol. Data are expressed as mean ± SD (n = 6). **(B)**. HeLa cells were treated with various concentrations of pep-CPDs (right panel) for 3, 6, 12, and 24 h. The cell viability was measured by the MTT assay. Data are expressed as mean ± SD (n = 6) **(C)**. Confocal images of the intracellular S100-β in the control group/S100-β (-) and S100-β (+) group (scale bar = 100 μm). Multicolor fluorescent cell imaging of HeLa cells incubated with pep-CPDs with S100-β and without in CLSM images, optical images, and merged images.

Before applying pep-CPDs to bioimaging experiments, the pep-CPDs’ influence on HeLa cells was assessed by LDH and MTT assays at various pep-CPD concentrations. Data are expressed as mean ± SD (*n* = 6). As shown in Figure 3A, the LDH assay was performed to evaluate the toxicity of the pep-CPDs toward the HeLa cells and results showed that there was lower toxicity even up to 1,000 ng/ml of the pep-CPDs. Figure 3B showed that the cells exhibited excellent viability in the presence of pep-CPDs during 4 h of culture, with >85% cell viability at a pep-CPD concentration of up to 1,000 ng/ml.

The fluorescent images from Figure 3C displayed the HeLa cells incubated with pep-CPDs with S100-β and without, under bright field and 480 nm excitation, respectively. Compared with bright red fluorescence observed in the absence of S100-β, there is weaker red fluorescence in the addition of S100-β, indicating that the presence of S100-β causes the fluorescence-quenching of the pep-CPDs in cells. Thus, we concluded that pep-CPDs can act as a valuable probe for S100-β detection in cellular imaging.

### Living C57 Mice Imaging With Pep-Carbon Polymer Dots

**FIGURE 4:**
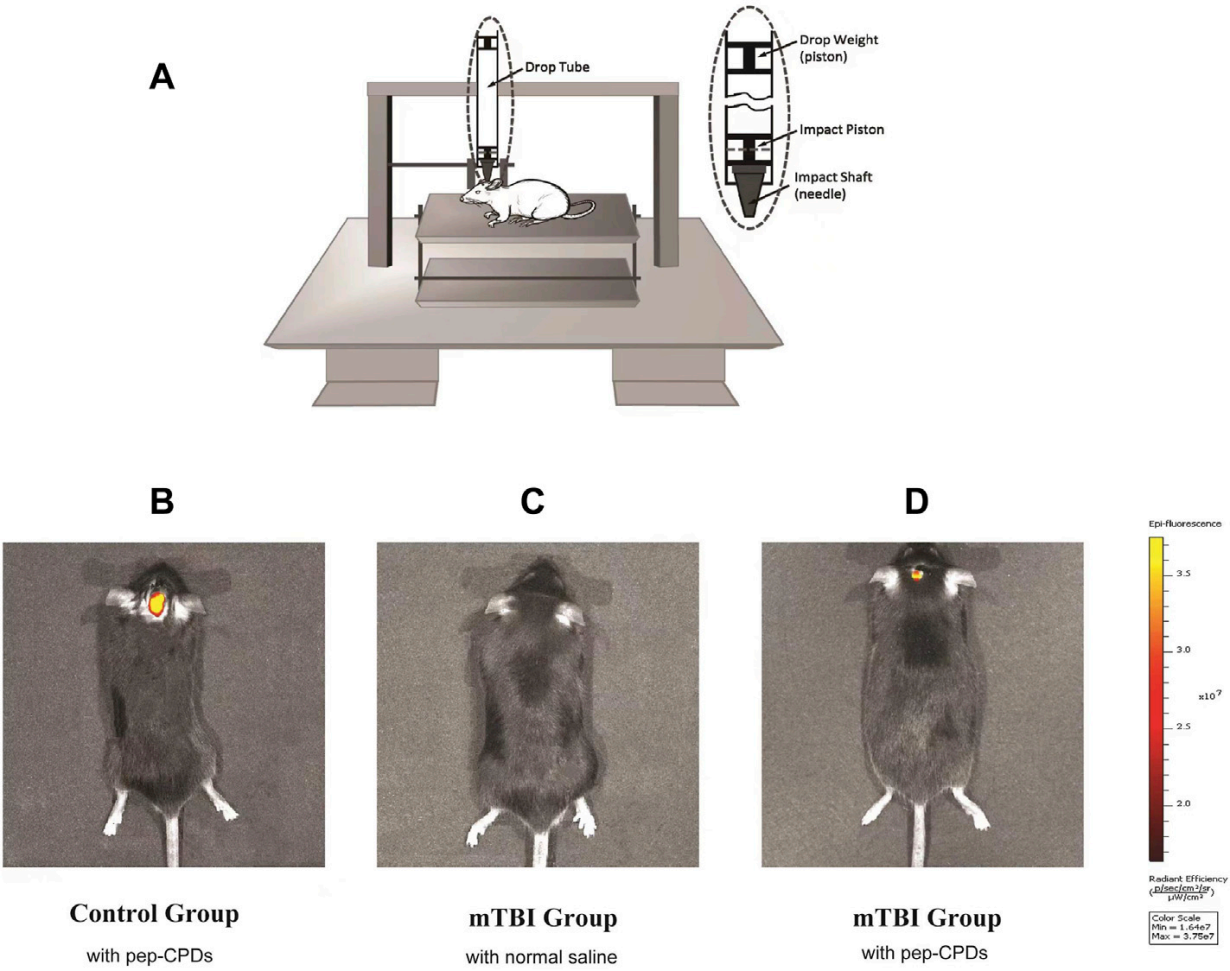
**(A)**. Modeling of mTBI using an improved weight-drop impactor. Fluorescence imaging of control group mouse **(B)** and mTBI group mouse **(C,D)**. *In vivo* fluorescence of the nanoprobe when injected *via* mouse inner canthus veniplex.

It was original to combine the construct mTBI model and fluorescent nanoprobe to operate the small animals in an *in vivo* imaging experiment. Firstly, to establish the brain injury animal model, we utilized an improved weight-drop impactor to induce mTBI (Figure 4A). NSSs were measured by the NSS and beam balance tests (Supplementary Table S1). The motor tests (normal = 0; for mTBI, the range is 1–2) and beam balance tests (normal = 0; for mTBI the range is 2–3) were considered comprehensively to characterize the mTBI (Flierl et al., 2009). After the evaluation, 39 mice were eligible for the mTBI inclusion criteria. The score range for the mTBI mice is shown in Supplementary Table S2. Further, in considering the most appropriate sex, age, and body weight of mice, we randomly chose 16 male mice (6–8 weeks old, approximately 25.1 ± 2.0 g body weight) from 25 mTBI mice modeling by the CCI machine for *in vivo* imaging. After the inner canthus injection of 150 μl pep-CPDs or normal saline, the mice were then anesthetized with diethyl ether for the subsequent imaging experiments. Three groups on the brain of mice received the following treatment: (A) control group (without injury) injected with pep-CPDs; (B) mTBI group injected with saline; and (C) mTBI group injected with pep-CPDs, respectively. Figure 4B has shown that no prominent fluorescence emission was found on the head from the mTBI group injected with only normal saline, indicating the negligible background fluorescence. Compared with the exhibited strong fluorescence signal of the control group (Figure 4D), the mTBI group treated with pep-CPDs displayed weaker fluorescence signal (Figure 4C)**,** which indicated that the probe pep-CPDs combined with the target biomarker S100-β can distinguish the mTBI group and control group. Therefore, the dynamic levels of S100-β protein can be observed *via in vitro* imaging.

### Acute Toxicity Testing Test With Body Weight Change Trends of Mice

Acute toxicity studies were conducted to evaluate the effects of pep-CPDs in a weight-of-the-evidence analysis. The long-term body weight changes of the mice were carried out by continuously monitoring the body weight of the control group (injected with 150 μl normal saline, *n* = 10) and the experimental groups (injected with 150 μl of 1,000, 500, and 100 μg/ml pep-CPDs, n = 10) under the same culture conditions for 28 days. The body weight of the mice in each group was weighed every 4 days. During the experiment process, no death was seen at the highest dose in both control and experimental groups (Supplementary Figure S3). Besides, the body weights of the experimental group retained the same growth trend as the control group. The experimental result showed that the comprehensive toxicity of pep-CPDs is suggested to be acceptable.

### Application in the Analysis of Clinical Mild Traumatic Brain Injury Instances Samples

**FIGURE 5:**
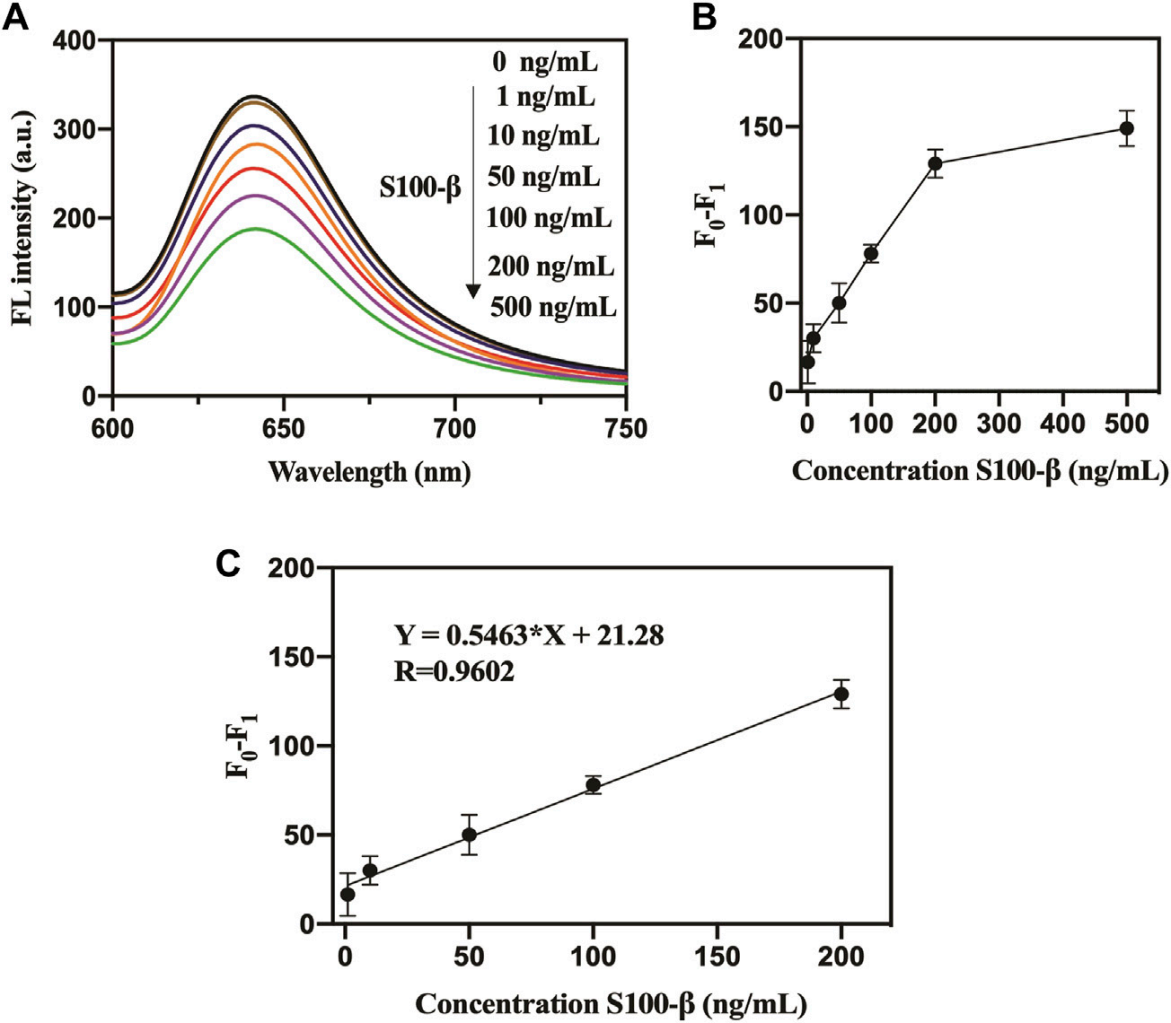
**(A)**. Fluorescence response of pep-CPD nanoprobe toward S100-β in serum. **(B)**. The relationship between the amounts of S100-β and the relative fluorescence intensity. **(C)**. Linear relationship between the relative fluorescence intensity of pep-CPDs and different concentrations of S100-β in serum. F0 and F1 represent the fluorescence intensity of the pep-CPDs in the addition of S100-β and without.

A rapid and straightforward detection approach is of fundamental importance in the examination of suspected mTBI with medico-legal importance (Thelin et al., 2013). Herein, this proposed assay was further used to detect the mTBI biomarker S100-β in serum samples. Quantitatively, the fluorescence signals and the target S100-β suggested a favorable linear relationship with an LOD of 0.3 ng/ml based on a signal-to-noise ratio (S/N = 3) (Figure 5). As shown in the Supplementary Table S3, the detection of the analytical performance of the nanoprobe toward the target S100-β was compared with previous works. The LOD analyzed by this method was lower than the LOD obtained in the previous literature (Han et al., 2020), and detection range was wider than the previous work (Wang et al., 2018). Besides, different amounts of S100-β were added to the human serum samples and then analyzed (Supplementary Table S3). The detection recoveries of human serum samples were obtained in the range of 96%–104% with a relative standard deviation (RSD) of 1.0%–1.5%, which confirmed the precise accuracy and good reproducibility of this assay for the determination of S100-β.

The concrete value of the concentrations of S100-β protein was of significance in predicting the outcome of clinical patients who suffer the mTBI (Liu et al., 2015). Herein, we compared the ELISA analysis and our method to evaluate the efficiency of detecting the S100-β levels in the serum of mTBI patients and healthy individual controls. For the mTBI group, S100-β levels in the serum samples were detected based on the traditional ELISA analysis method and novel sensing method; both have similar tendency and consistency (Table 1). Furthermore, compared to the results by the ELISA, our sensing method was also able to sensitively detect the S100-β levels in the control group (0.9 ± 1.9 ng/ml), in which the levels of S100-β were in the range of average values (Oris et al., 2018). This experiment further demonstrated the usefulness of protein S100-β as a biomarker in mTBI patients. Accordingly, this novel method based on pep-CPDs offers many benefits in terms of sensitivity, specificity, accuracy, and practicability, having advantages over the ELISA and other traditional detection methods for identifying mTBI in forensic and clinical laboratory diagnostics, which will likely become one of the most optimum candidates for rapid and safe detection methods for mTBI patients (Li et al., 2020; Rickard et al., 2020; Krausz et al., 2021; Ngowi et al., 2021).

**TABLE 1:** mTBI Patients' group characteristics comparison using biosensor analysis and ELISA.

	mTBI group (N = 10)	Control group (N = 10)	P-value
Sex (M/F)	5/5	5/5	N/A
Age (years)	23.6 ± 4.3	22.1 ± 5.2	0.42
Height (cm)	166.4 ± 11.3	169.3 ± 9.6	0.55
Weight (kg)	68.2 ± 9.9	67.3 ± 7.6	0.86
Mechanism of injury	Traffic accident (5)	N/A	N/A
GCS score	Falling (4)	N/A	N/A
Loss of consciousness (min)	Sport activity (1)	N/A	N/A
S100-β levels (this analysis)	14 (6)	0.9 ± 1.9	—
S100-β levels (ELISA analysis)	13 (4)	2.5 ± 0.6	—
	< 30 min	—	—
	22.8 ± 5.6	—	—
	23.7 ± 3.2	—	—

## Conclusion

In summary, we successfully demonstrated the application of the pep-CPDs’ *in vitro* and *in vivo* visualization of S100-β. The nanoprobe exhibited high selectivity and sensitivity for mTBI biomarker S100-β detection even in complex biological systems, as well as good favorable properties in bioimaging for a weight drop-induced mTBI mouse model. The described versatile nanoprobe approach, demonstrated in the *in vivo* imaging of the animal model and rapid detection of S100-β in clinical instances, has implications for clinical diagnostics and practical applications. Meanwhile, limitations such as further safety evaluation, long-term toxicity studies as well as the effectiveness in more clinical and forensic cases require more investigations in the future.

## Data Availability

The original contributions presented in the study are included in the article/Supplementary Material, further inquiries can be directed to the corresponding authors.
