# Supplementary figures and images for: A Versatile Pep-CPDs Nanoprobe for Rapid Detection of mTBI Biomarker in Clinical Instances and Safe Fluorescence Imaging In Vivo for Improved Weight-Drop Mouse Model
Source: Front Bioeng Biotechnol. 2022 Mar 7;10:807486. doi: 10.3389/fbioe.2022.807486 (PMC8942774; doi:10.3389/fbioe.2022.807486)

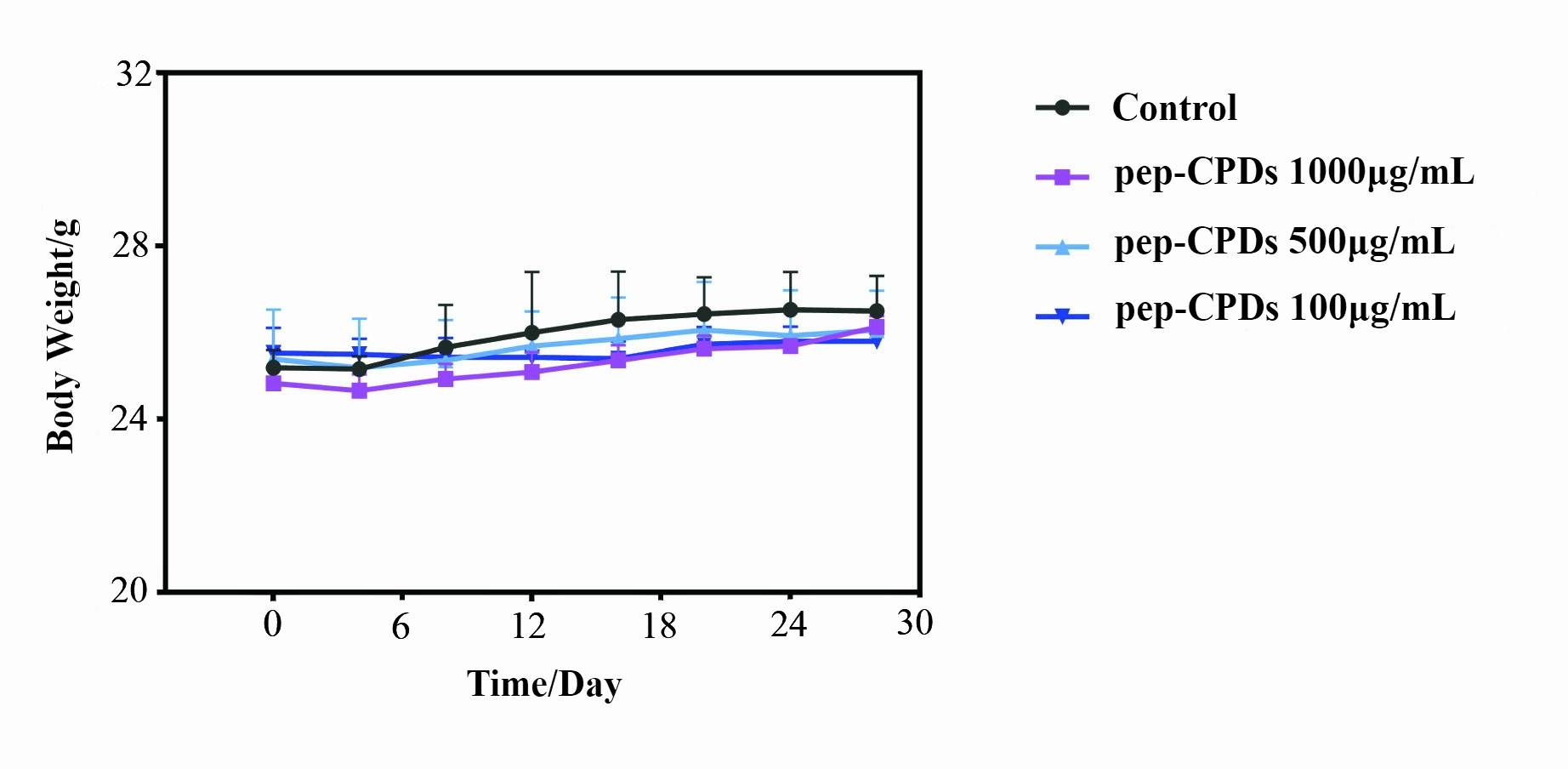

Supplement: Supplementary file 1 [file Image3.JPEG]

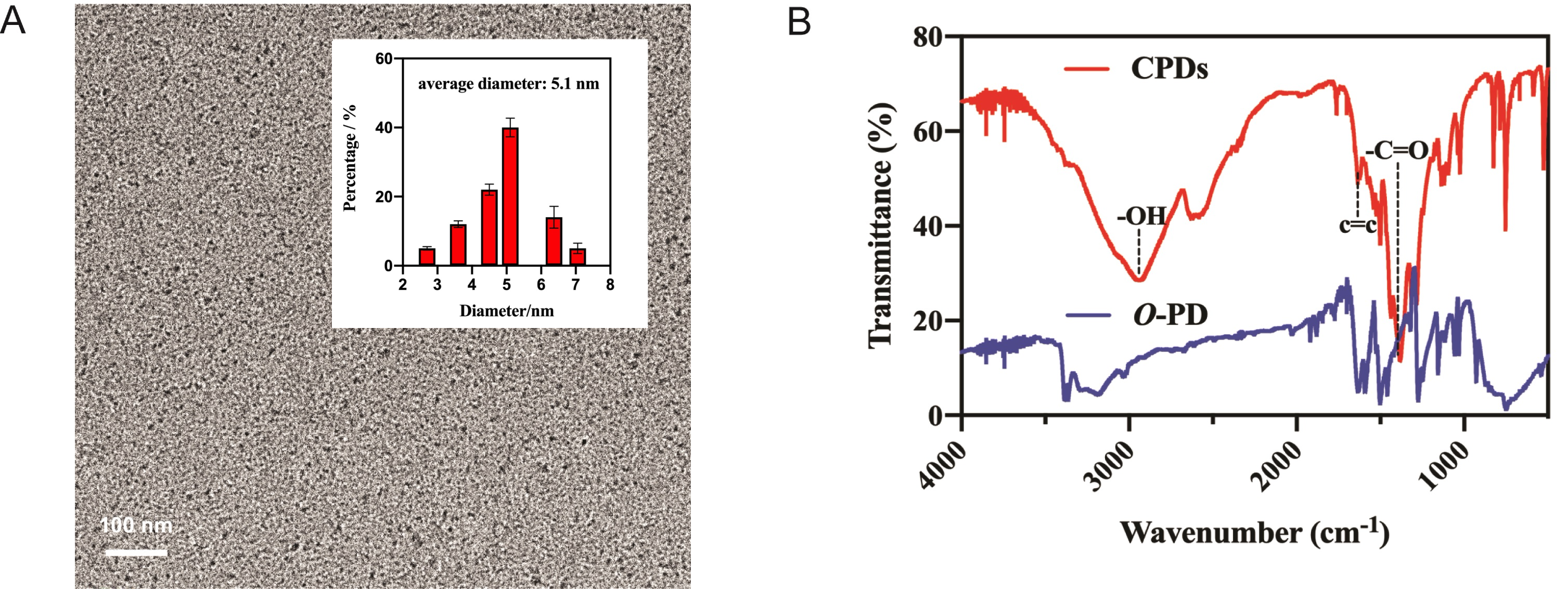

Supplement: Supplementary file 2 [file Image1.JPEG]

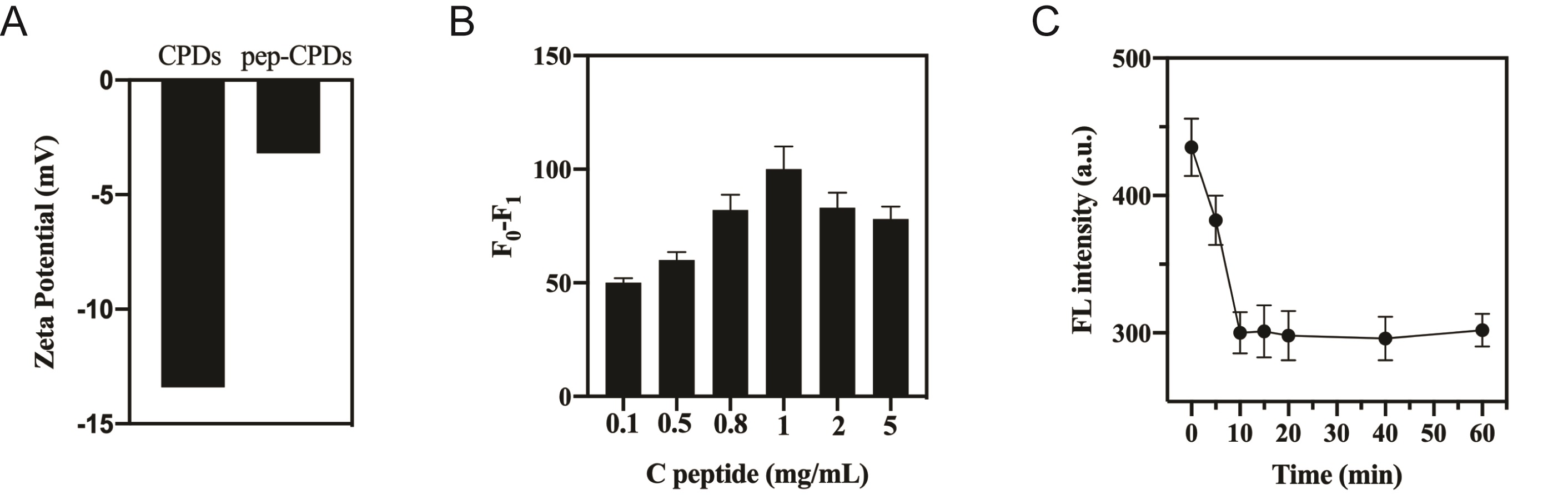

Supplement: Supplementary file 3 [file Image2.JPEG]
